# [S-methyl-^11^C]-L-methionine positron emission tomography/computed tomography imaging parameters to evaluate early response for esophageal cancer with neoadjuvant carbon ion radiotherapy

**DOI:** 10.1038/s41598-022-17962-x

**Published:** 2022-08-11

**Authors:** Kazuo Narushima, Ryuichi Nishii, Shinichi Okazumi, Hideaki Shimada, Yasunori Akutsu, Takamasa Maeda, Shigeo Yasuda, Shigeru Yamada, Kiyohiko Shuto, Kentaro Tamura, Kana Yamazaki, Makoto Shinoto, Hitoshi Ishikawa, Mikito Mori, Hisahiro Matsubara

**Affiliations:** 1 Department of Surgery, Secomedic Hospital, Chiba, Japan; 2 Department of Frontier Surgery, Chiba University Graduate School of Medicine, Chiba, Japan; 3 Quantum Life and Medical Science Directorate, National Institutes for Quantum Science and Technology (QST), QST Hospital, Chiba, Japan; 4 Department of Molecular Imaging and Theranostics, Institute for Quantum Medical Science, Quantum Life and Medical Science Directorate, National Institutes for Quantum Science and Technology (QST), Chiba, Japan; 5 Department of Surgery, Toho University Sakura Medical Center, Chiba, Japan; 6 Department of Gastroenterological Surgery, Toho University Graduate School of Medicine, Tokyo, Japan; 7 Miwa Central Clinic, Ibaraki, Japan; 8 Department of Radiology, Chiba Rosai Hospital, Chiba, Japan; 9 Department of Surgery, Teikyo University Chiba Medical Center, Chiba, Japan

**Keywords:** Cancer imaging, Oesophageal cancer, Radiotherapy

## Abstract

This study aimed to evaluate the uptake of the clinical effectiveness of [S-methyl-^11^C]-L-methionine positron emission tomography/computed tomography (MET PET/CT) in patients with esophageal cancer and to investigate MET PET/CT imaging parameters to assess early response for esophageal cancer with neoadjuvant carbon ion radiotherapy (CIRT). MET PET/CT scans were performed in nineteen patients before and 3 weeks after completion of CIRT. After Surgery, the effect of neoadjuvant CIRT was investigated by examining the relationship between each parameter of MET uptake and the histological assessment (grade and tumor residual ratio). Four parameters of MET uptake were the maximum and minimum standardized uptake values of pre and post CIRT (pre-SUVmax, pre-SUVmean, post-SUVmax, and post-SUVmean). MET PET/CT imaging of esophageal cancer was clearly demonstrated. The post-SUVmax was the most suitable parameter. When the cutoff value was set as post-SUVmax = 6.21, the sensitivity, the specificity, and the accuracy of Grades 3 were 100.0%, 63.6%, and 78.9%, respectively. And there was a positive relationship between the tumor residual ratio and post-SUVmax (*R*^*2*^ = *0.38, p* < *0.005*). MET PET/CT is clinically useful for the assessment of early response to neoadjuvant CIRT in esophageal cancer. Particularly, post-SUVmax is considered a promising PET imaging parameter.

## Introduction

Esophageal cancer is the sixth leading cause of cancer mortality worldwide^1^ and has a poor prognosis, with a 5-year relative survival rate of 15–25%^2^. The most typical curative treatment for esophageal cancer is surgery; however, in Japan, neoadjuvant chemotherapy is recommended to improve prognosis^3,4^. Regarding other neoadjuvant therapies, the efficacy of neoadjuvant chemoradiotherapy combined with radiation therapy has also been reported^5,6^.

Since 1994, QST Hospital (former National Institute of Radiological Sciences [NIRS] Hospital) has been providing carbon ion radiotherapy (CIRT)^7^. Carbon ion beams have a high relative biological effectiveness with a high linear energy transfer and are capable of creating a very high dose peak, which is called a Bragg peak. Considering these features, CIRT is more effective and causes less radiation damage to normal tissues than conventional radiotherapy. From 2004 to 2008, the world’s first phase I/II clinical trial of neoadjuvant CIRT for esophageal cancer was conducted in collaboration with the Department of Frontier Surgery, Graduate School of Medicine, Chiba University. CIRT has a significantly good therapeutic effect with a complete response (CR) of 38.7%, despite the fact that CIRT is a radiation therapy alone and has a short-term irradiation of eight times^8^.

For neoadjuvant therapy of esophageal cancer, the usefulness of 2-[^18^F]fluoro-2-deoxy-D-glucose (FDG) positron emission tomography/computed tomography (PET/CT) in determining efficacy has been reported in many studies^9–13^. [S-methyl-^11^C]-L-methionine (MET), mainly transported by system L amino acid transporters^14,15^, has been clinically used as a tumor-seeking agent for PET imaging for several decades^16^. MET PET/CT has fewer false positives due to inflammation and necrosis than FDG PET/CT^17^. MET PET/CT may be more helpful than FDG PET/CT or other imaging modalities in determining the efficacy of radiotherapy because radiotherapy is more likely to be associated with necrosis and inflammation than chemotherapy. The usefulness of MET PET/CT in radiotherapy has been reported in the diagnosis of relapse in brain tumors^18^, and its efficacy in lung and head and neck cancers has been assessed^19–23^. However, the clinical effectiveness of MET PET/CT in esophageal cancer has not yet been evaluated.

This study aimed to evaluate the uptake of MET PET/CT in patients with esophageal cancer and to investigate MET PET/CT imaging parameters to assess early response for esophageal cancer with neoadjuvant CIRT.

## Methods

### Patient population

From August 2004 to June 2007, 24 consecutive patients with histologically proven thoracic esophageal squamous cell carcinoma who underwent curative esophagectomy with neoadjuvant CIRT based on the reported method^8^ were reviewed retrospectively. Four patients who did not undergo PET both pre- and post-CIRT were excluded. Moreover, another patient was excluded because the interval between the post-CIRT PET/CT scans and surgery was more than 30 days. In total, 19 (14 men and 5 women; median age, 63 [47–80] years) patients were included in this study. The study protocol was approved by QST (NIRS Hospital) Hospital, and all patients provided written informed consent. clinical staging before and after CIRT, including endoscopy, CT, endoscopic ultrasound (EUS), radiography of the upper digestive tract, and neck ultrasonography, was performed. According to these modalities, tumors were staged clinically based on the 11th edition of the Japanese Classification of Esophageal Cancer^24,25^.

### CIRT

CIRT was performed at QST Hospital. The CIRT protocol was based on the phase I/II clinical trial of neoadjuvant CIRT for esophageal cancer as previously reported [8]. As described shortly, CIRT is a radiation therapy alone without concurrent chemotherapy. The total dose ranged from 28.8 to 35.2 GyE, and single doses of 3.6 to 4.4 GyE were administered four times a week for eight times. External beam radiotherapy was delivered by two opposing anteroposterior and posteroanterior fields. The planning target volume was determined on CT scanning which included the whole primary tumor with a 3 cm margin in the craniocaudal direction and metastatic LNs with a minimum of a 1 cm margin. Consequently, all patients underwent radical esophagectomy at 4-week intervals after the completion of CIRT.

### Surgical therapy and histological response evaluation

Surgery was performed in all patients within 1 week of the last PET scan in Department of Frontier Surgery, Graduate School of Medicine, Chiba University. None of the patients showed local tumor progression or distant metastases during preoperative therapy. Surgical therapy consisted of transthoracic esophagectomy with three-field lymphadenectomy. Hematoxylin and eosin staining was performed for pathological evaluation, and all specimens were evaluated by two experienced pathologists who were blinded of the clinical and PET data. Tumor regressions were classified histologically based on the 11th edition of the Japanese Classification of Esophageal Cancer. Patients were divided into the following groups: Grade 1 (the disappearance rate of less than two-thirds in cancer cells), Grade 2 (the disappearance rate of more than two-thirds in cancer cells), and Grade 3 (the complete disappearance of cancer cells)^24,25^. Furthermore, the residual rate of cancer cells was quantified visually.

### MET PET/CT imaging and image analysis

#### PET/CT scan

Thirty-eight MET PET/CT scans were performed in all patients before and 3 weeks after the completion of CIRT. Twenty min after intravenous injection of 740 MBq (20 mCi) of MET, consecutively unenhanced CT scan was performed, followed by the emission images covering the area from the neck to the upper abdomen (four bed positions, 7 min duration each) were acquired for 30 min using a Biograph (Siemens Medical Systems, Nashville, TN, USA). The CT data were used for attenuation correction. The PET data sets were reconstructed using a filtered back-projection algorithm. Coregistered scans were displayed using the software (Fusion Workstation Siemens Medical Systems, Nashville, TN, USA).

#### Evaluation of MET uptake

Volumes of interest (VOIs) of the tumor were outlined on the co-registered CT image of each patient when the registration images of the target lesion were matched between PET and CT images. The maximum and mean standardized uptake values (SUVmax and SUVmean), which are convenient semiquantitative methods and most widely used, were assessed for MET uptake by the lesion. Both SUVmax and SUVmean were measured pre- and post-CIRT. MET uptake consists of four parameters (pre/post-SUVmax, pre/post-SUVmean). The effect of neoadjuvant CIRT was investigated by examining the relationship between each parameter of MET uptake and histological efficacy (grade and quantified tumor residual ratio).

### Statistical analyses

All values were calculated as means ± standard deviations. Statistical analyses were performed using SAS JMP statistical software version 15 (SAS Institute, Cary NC, USA), with *p* values of less than 0.05 considered statistically significant. MET uptakes of Grades 1, 2, and 3 were compared by performing the nonparametric Tukey‒Kramer honestly significant difference test. MET uptakes of Grades 1, 2, and 3 were compared by performing the Mann–Whitney U test. The cutoff values were determined using the receiver operating characteristic curve. The best cutoff points for balancing the sensitivity and specificity of a test are the point on the curve closest to the (0, 1) point. Optimal sensitivity and specificity are defined as those yielding the minimal value for (1 − sensitivity)^2^ + (1 − specificity)^2^^26^. Linear regression between residual tumor rate and SUV was calculated, and Spearman’s correlation between the two groups was analyzed.

### Ethics approval

All procedures and subsequent analyses were performed with the approval of the Institutional Review Boards of Quantum and Radiological Science and Technology (QST) (reference number: 21–003). All enrolled patients received explanations, and then they provided written informed consent regarding this clinical study. Our study was conducted in accordance with the principles outlined in the 1964 Declaration of Helsinki and its later amendments.

## Results

All 19 patients were diagnosed with squamous cell carcinoma, and the depths of tumor invasion were T1, T2, and T3 in 7, 7, and 5 patients, respectively (Table 1).

**Table 1:** Summary of patient profiles and MET PET/CT findings in patients with neoadjuvant CIRT for esophageal cancer.

Patient	Age (y)	Sex	Tumor location	Depth of tumor invasion (T)	Tumor size (mm)	Clinical stage	Preoperative radiotherapy (GyE)	Histological effect (grade)	Residual tumor rate (%)	SUVmax		SUVmean	
										pre	post	pre	post
1	47	M	Mt	1b	25	I	28.8	2	10	6.38	4.88	6.65	4.58
2	63	F	Mt	1b	40	I	28.8	2	1	4.98	3.16	5.50	4.12
3	71	F	Mt	3	60	III	28.8	1b	40	8.12	5.54	9.48	4.69
4	63	M	Mt	2	40	II	28.8	2	10	8.53	5.44	6.01	3.79
5	69	M	Mt	1b	50	I	28.8	3	0	7.48	4.42	5.55	3.25
6	64	M	Mt	1b	50	I	30.4	3	0	8.64	5.06	6.02	3.64
7	62	M	Lt	3	40	II	30.4	2	1	12.84	7.25	8.31	4.63
8	71	M	Lt	2	50	II	32	3	0	6.85	3.98	5.91	4.21
9	51	M	Mt	2	40	II	32	1b	60	12.56	7.52	9.38	5.88
10	63	M	Lt	1b	40	II	32	2	20	10.33	5.31	10.14	4.91
11	71	M	Lt	3	55	III	32	2	5	7.51	4.37	7.21	4.19
12	59	M	Lt	2	60	II	33.6	3	0	7.98	4.69	5.29	3.02
13	80	F	Mt	2	40	II	33.6	3	0	10.41	5.40	6.21	3.52
14	64	M	Mt	2	30	III	33.6	3	0	9.26	5.50	5.69	4.14
15	57	F	Mt	1b	20	I	35.2	2	2	6.44	4.24	4.00	2.81
16	71	M	Ut	3	40	II	35.2	2	3	7.73	4.86	4.94	3.01
17	63	M	Mt	3	50	II	35.2	2	5	7.15	4.58	6.40	3.79
18	60	F	Lt	1b	15	I	35.2	3	0	4.08	3.20	3.42	2.78
19	67	M	Lt	2	40	III	35.2	3	0	10.30	5.71	5.74	3.82
Average										8.29	6.41	5.01	3.94
SD										2.28	1.79	1.11	0.80

*Ut*, upper thoracic esophagus; *Mt*, middle thoracic esophagus; *Lt*, lower thoracic esophagus.

### MET uptake in esophageal cancer lesion

When the lesion uptake of MET was compared with depth of tumor invasion, pre-SUVmax were 6.90 ± 2.13, 9.41 ± 1.88, and 8.67 ± 2.36 for T1, T2, and T3, respectively (*n.s.* for the difference between T1 and T2, *n.s.* for T1 and T3, *n.s.* for T2 and T3). The pre-SUVmean were 4.33 ± 0.86, 5.46 ± 1.09, and 5.33 ± 1.17 for T1, T2, and T3, respectively (*p* < *0.05* for T1 and T2, *n.s.* for T1 and T3, *n.s.* for T2 and T3).

### Relationship between MET uptake of pre-CIRT and post-CIRT

A summary of each case is described in Table 1. When pre-SUV (uptake of pre-CIRT) was compared with postSUV (uptake of post-CIRT), SUVmax were 8.29 ± 2.28 and 6.41 ± 1.79 for pre-SUVmax and post-SUVmax, respectively (*p* < *0.005* for the difference between pre-SUVmax and post-SUVmax). Meanwhile, SUVmean were 5.01 ± 1.11 and 3.94 ± 0.80 for pre-SUVmean and post-SUVmean, respectively (*p* < *0.005* for pre-SUVmean and post-SUVmean).

### Relationship between MET uptake and all grades

**Figure 1:**
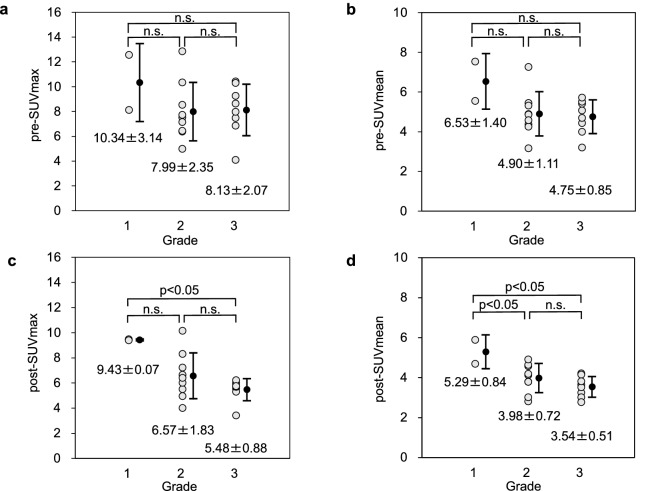
PET imaging parameters of MET uptake and all grades. There was no significant difference in the pre-SUVmax among all grades (**a**). There was also no significant difference in the pre-SUVmean among all grades (**b**). There was a significant difference in post-SUVmax between Grades 1 and 3 (*p* < *0.05*) (**c**). There was also a significant difference in post-SUVmax between Grades 1 and 2 (*p* < *0.05*) and Grades 1 and 3 (*p* < *0.05*) (**d**). The Tukey‒Kramer honestly significant difference test was used for statistical analysis.

The relationship between MET uptake and all grades is shown in Fig. 1. When the lesion uptake of MET was compared with all grades, pre-SUVmax were 10.34 ± 3.14, 7.99 ± 2.35, and 8.13 ± 2.07 for Grades 1, 2, and 3, respectively (*n.s.* for the difference between Grades 1 and 2, *n.s.* for Grades 1 and 3, *n.s.* for Grades 2 and 3; Fig. 1a). The pre-SUVmean were 6.53 ± 1.40, 4.90 ± 1.11, and 4.75 ± 0.85 for Grades 1, 2, and 3, respectively (*n.s.* for Grades 1 and 2, *n.s.* for Grades 1 and 3, *n.s.* for Grades 2 and 3; Fig. 1b). The post-SUVmax were 9.43 ± 0.07, 6.57 ± 1.83, and 5.48 ± 0.88 for Grades 1, 2, and 3, respectively (*n.s.* for Grades 1 and 2, *p* < *0.05* for Grades 1 and 3, *n.s.* for Grades 2 and 3; Fig. 1c). Meanwhile, post-SUVmean were 5.29 ± 0.84, 3.98 ± 0.72, and 3.54 ± 0.51 for Grades 1, 2, and 3, respectively (*p* < *0.05* for Grades 1 and 2, *p* < *0.05* for Grades 1 and 3, *n.s.* for Grades 2 and 3; Fig. 1d).

### Relationship between MET uptake and Grades 1, 2, and 3

**Figure 2:**
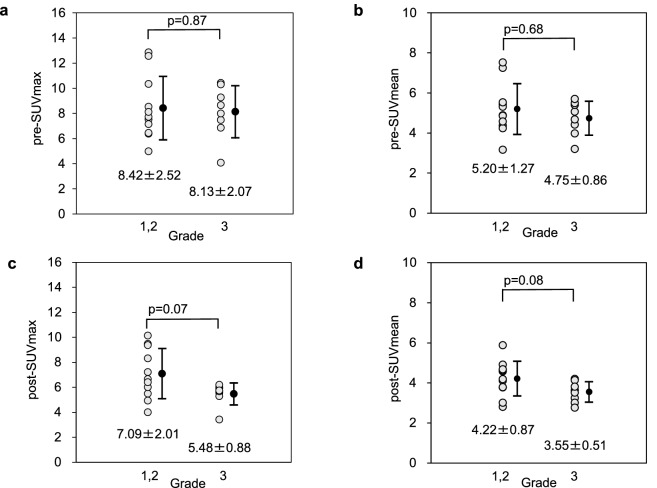
Relationship between MET and Grades 1, 2, and 3. There was no significant difference in PET imaging parameters among Grades 1, 2, and 3. (**a**) pre-SUVmax. (**b**) pre-SUVmean. (**c**) post-SUVmax. (**d**) post-SUVmean. The Mann–Whitney U test was used for statistical analysis.

The relationship between MET uptake and Grades 1, 2, and 3 are shown in Fig. 2. In the comparison between the uptake of Grades 1, 2, and 3, pre-SUVmax were 8.42 ± 2.52 and 8.13 ± 2.07 for Grades 1 and 2 and Grade 3, respectively (*p* = *0.87*, difference between Grades 1, 2, and 3; Fig. 2a). The pre-SUVmean were 5.20 ± 1.27 and 4.75 ± 0.86 for Grades 1 and 2 and Grade 3, respectively (*p* = *0.68*, Grades 1, 2, and 3, respectively; Fig. 2b). The post-SUVmax were 7.09 ± 2.01 and 5.48 ± 0.88 for Grades 1 and 2 and Grade 3, respectively (*p* = *0.07* for Grades 1, 2 and 3; Fig. 2c). The post-SUVmean were 4.22 ± 0.87 and 3.55 ± 0.51 for Grades 1 and 2 and Grade 3, respectively (*p* = *0.08*; Grades 1, 2, and 3; Fig. 2d).

### Differential diagnosis in terms of grade 1, 2, and 3 by MET uptake

The accuracy of the differential diagnosis between the uptake of Grades 1, 2, and 3 is summarized in Table 2. When the cutoff value was set as pre-SUVmax 7.48, the sensitivity, specificity, and accuracy were 37.5%, 63.6%, and 52.6%, respectively, whereas if the cutoff value was set as pre-SUVmean 4.69, the sensitivity, specificity, and accuracy were 50.0%, 63.6%, and 57.9%, respectively. When the cutoff value was set as post-SUVmax 6.21, the sensitivity, specificity, and accuracy were 100.0%, 63.6%, and 78.9%, respectively, whereas if the cutoff value was set as post-SUVmean 3.64, the sensitivity, specificity, and accuracy were 62.5%, 81.8%, and 73.7%, respectively.

**Table 2:** Diagnostic accuracy of PET imaging parameter between MET uptake of Grades 1, 2, and 3. The cutoff values for pre-SUVmax, pre-SUVmean, post-SUVmax, and post-SUVmean were 7.48, 4.69, 6.21, 3.64, respectively.

	Sensitivity	Specificity	PPV	NPV	Accuracy
Pre-SUVmax	37.5	63.6	42.9	58.3	52.6
Pre-SUVmean	50.0	63.6	50.0	63.6	57.9
Post-SUVmax	100.0	63.6	66.7	100.0	78.9
Post-SUVmean	62.5	81.8	71.4	75.0	73.7

*PPV*, positive predictive value; *NPV*, negative predictive value.

### Correlation between residual tumor rate and MET uptake

**Figure 3:**
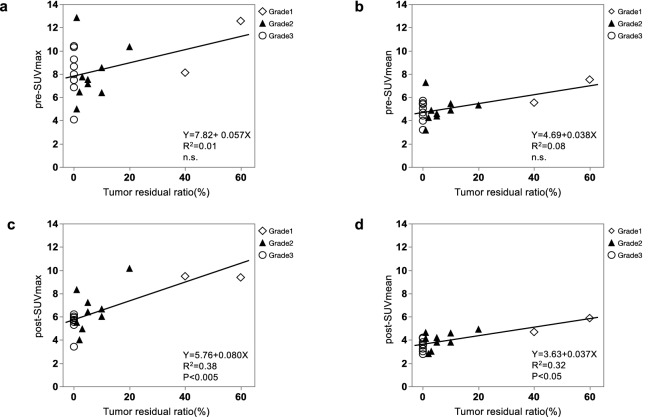
Correlation between residual tumor rate and PET imaging parameters of MET uptake. There was a positive linear relationship between the residual tumor rate and all the parameters. (**a**) pre-SUVmax. (**b**) pre-SUVmean. (**c**) post-SUVmax. (**d**) post-SUVmean. Spearman’s test was used for the statistical analysis.

The correlation between the residual tumor rate and uptake of MET is shown in Fig. 3. A positive linear relationship between the residual tumor rate and pre-SUVmax (Y = 7.82 + 0.057 X, *R*^*2*^ = *0.01*, *correlation coefficient* = *0.10*, *n.s.*; Fig. 3a), a positive relationship between the residual tumor rate and pre-SUVmean (Y = 4.69 + 0.038*X*, *R *^*2*^ = *0.08*, *correlation coefficient* = *0.29, n.s.*; Fig. 3b), a positive relationship between the residual tumor ratio and post-SUVmax (Y = 5.76 + 0.080*X*, *R*^*2*^ = *0.38*, *correlation coefficient* = *0.62, p* < *0.005*; Fig. 3c), and a positive relationship between the residual tumor rate and post-SUVmean (Y = 3.63 + 0.037*X*, *R*^*2*^ = *0.32*, *correlation coefficient* = *0.56, p* < *0.05*; Fig. 3d) were observed.

**Figure 4:**
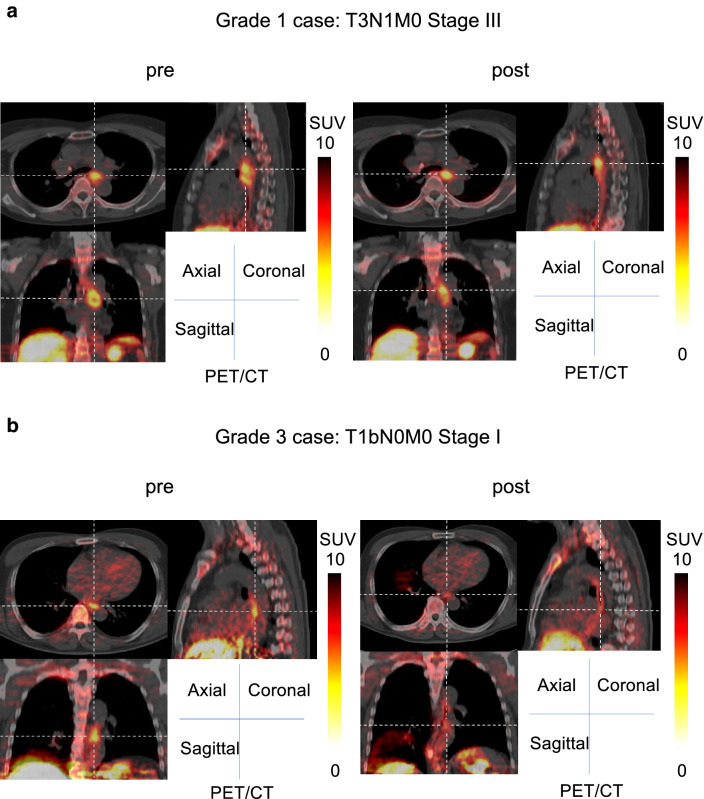
Representative cases. (**a**) Grade 1 cases: T3N1M0, stage III. (**b**) Grade 3 cases: T1bN0M0, stage I.

Representative cases of MET PET/CT images for Grade 1 cases: T3N1M0, stage III and Grade 3 cases: T1bN0M0, stage I are shown in Fig. 4.

## Discussion

This study investigated the uptake of MET in esophageal cancer and its early response in neoadjuvant CIRT of esophageal cancer and its relationship with MET uptake on MET PET/CT.

First, MET PET/CT imaging of esophageal cancer was clearly demonstrated. In terms of depth of tumor invasion, there was a tendency for the accumulation to be higher in T2 and T3 than in T1.

In CIRT of esophageal cancer, MET uptake clearly decreased in response to CIRT. These findings are the first report of MET PET/CT in esophageal cancer. MET PET/CT was as useful as the reported regarding FDG PET/CT^8–12^. In some cases, low uptake of MET was observed on the post-CIRT images, which were proved by the endoscopic examination after CIRT. Although MET PET/CT is considered to have fewer false positives due to inflammation than FDG PET/CT, there is also some degree of MET uptake into the inflammatory site^17^.

Chemoradiotherapy is as important as surgery in the treatment of esophageal cancer^27–29^. In radical chemoradiotherapy for stage II–III esophageal cancer, the CR rate was 70.6%, and 84% of them were non-recurrences^29^. Patients with CR after chemoradiotherapy are expected to have a relatively good prognosis without surgery.

In CIRT, there is a possible treatment option to avoid surgery in patients with CR. Therefore, it is essential to preoperatively diagnose Grade 3 cases after CIRT. In addition, neoadjuvant chemoradiotherapy had significantly higher rates of morbidity and mortality after surgery than neoadjuvant chemotherapy^30^. The advantage of avoiding surgery after radiotherapy is of paramount importance.

The timing of MET PET/CT to diagnose histological efficacy was considered to be more useful approximately 3 weeks after CIRT, that is, just before or several weeks before surgery than before CIRT. Since the histological efficacy is determined from the surgical specimens after CIRT, it seems reasonable that MET uptake after CIRT close to the surgery best reflects the histological efficacy.

The post-SUVmax was the most suitable parameter for the diagnosis of Grade 3, because the diagnostic accuracy of Grade 3 was the highest for post-SUVmax at approximately 80%.

In this study, we attempted to quantify the histological efficacy as the residual tumor rate and to examine the histological efficacy in detail. When the correlation between residual tumor rate and MET uptake was examined, a positive linear relationship was observed for post-SUVmax, followed by the parameter using post-SUVmean.

Since the residual tumor rate represents the density of the tumor, SUVmean is a theoretically ideal parameter for reflecting tumor activity. However, in this study, the prediction of residual tumor rate using SUVmean was found to be inferior to that using SUVmax. The issue of measurement accuracy in the SUVmean method was considered to be the reason for this. For a more accurate quantitative evaluation using SUVmean, it is essential to accurately determine the margins of the lesion. However, the CT component of PET/CT is non-contrast imaging, making it difficult to accurately determine the margin of the tumor lesion. In addition, because the esophagus is a luminal organ, semiquantitative evaluation is affected by the uptake value of the air portion of the esophageal lumen. Since the method used in the current study was a manual setting of the VOIs, it is not reproducible. Considering the reasons described above, the evaluation method using SUVmax is considered to be more appropriate and practical than that using SUVmean.

If the residual tumor rate can be diagnosed, various treatment options are possible. If residual tumor rates are minimal, additional chemotherapy or chemoradiation for Grade 3 can be selected. If the residual tumor rates are low, less invasive surgical treatment, such as omitting cervical lymph node dissection, can be considered. If residual tumor rates are high, preoperative treatment may not be sufficiently effective, but other preoperative treatments can be added or modified. When residual tumor rates are high, the efficacy of preoperative treatment may be uncertain, but other treatments can be added or modified to improve the treatment effect.

As described above, in CIRT for esophageal cancer, it was possible to diagnose local effects, such as tumor disappearance and tumor persistence, in detail by devising the timing of measurement and analysis method of MET accumulation. MET may be a useful imaging marker for the selection of other therapeutic options.

This study has some limitations. First, the number of patients analyzed was small due to the number of cases set in this clinical trial. Second, since there were only two Grade 1 cases, further study is needed for a detailed comparison with histological effects. Third, prognostic analysis was not performed. In this clinical trial study protocol with limited number of patients and longer observation period, we are planning to determine clear indication criteria of CIRT for esophageal cancer by a combination of MET PET with other important prognostic factors.

## Conclusion

This is the first study to report the relationship between esophageal cancer and MET accumulation, and according to this study, MET PET/CT is feasible for the imaging of esophageal cancer. Furthermore, MET PET/CT is clinically useful for the assessment of early response to neoadjuvant CIRT in esophageal cancer. In particular, post-SUVmax in MET PET/CT is considered a promising PET imaging parameter.

## Data Availability

The datasets used and/or analyzed during the current study are available from the corresponding author on reasonable request.
